# Supplementary material for: A SPLICS reporter reveals α-synuclein regulation of lysosome-mitochondria contacts which affects TFEB nuclear translocation
Source: Nat Commun. 2024 Feb 19;15:1516. doi: 10.1038/s41467-024-46007-2 (PMC10876553; doi:10.1038/s41467-024-46007-2)

Supplementary Information for

A SPLICS reporter reveals α-synuclein regulation of lysosome-mitochondria contacts which affects TFEB nuclear translocation

Flavia Giamogante[1,#], Lucia Barazzuol[1,#], Francesca Maiorca[2], Elena Poggio[2], Alessandra Esposito[3,4], Anna Masato[2,5], Gennaro Napolitano[3,4], Alessio Vagnoni[6], Tito Calì[1,7,8] * and Marisa Brini[2,8,9] *

[1] Department of Biomedical Sciences (DSB), University of Padova, Italy
[2] Department of Biology (DIBIO), University of Padova, Italy
[3] Telethon Institute of Genetics and Medicine (TIGEM), Naples, Italy.
[4] Department of Medical and Translational Science, Federico II University, Naples, Italy.
[5] UK-Dementia Research Institute at UCL, University College London, London, UK.
[6] Department of Basic and Clinical Neurosciences, Maurice Wohl Clinical Neuroscience Institute, Institute of Psychiatry, Psychology and Neuroscience, King's College London, London, UK
[7] Padova Neuroscience Center (PNC), University of Padova, Padova, Italy
[8] Study Center for Neurodegeneration (CESNE), University of Padova, Italy
[9] Department of Pharmaceutical and Pharmacological Sciences (DSF), University of Padova, Italy
[#] These authors contributed equally

* corresponding author: marisa.brini@unipd.it; tito.cali@unipd.it

Supplementary Figure 1

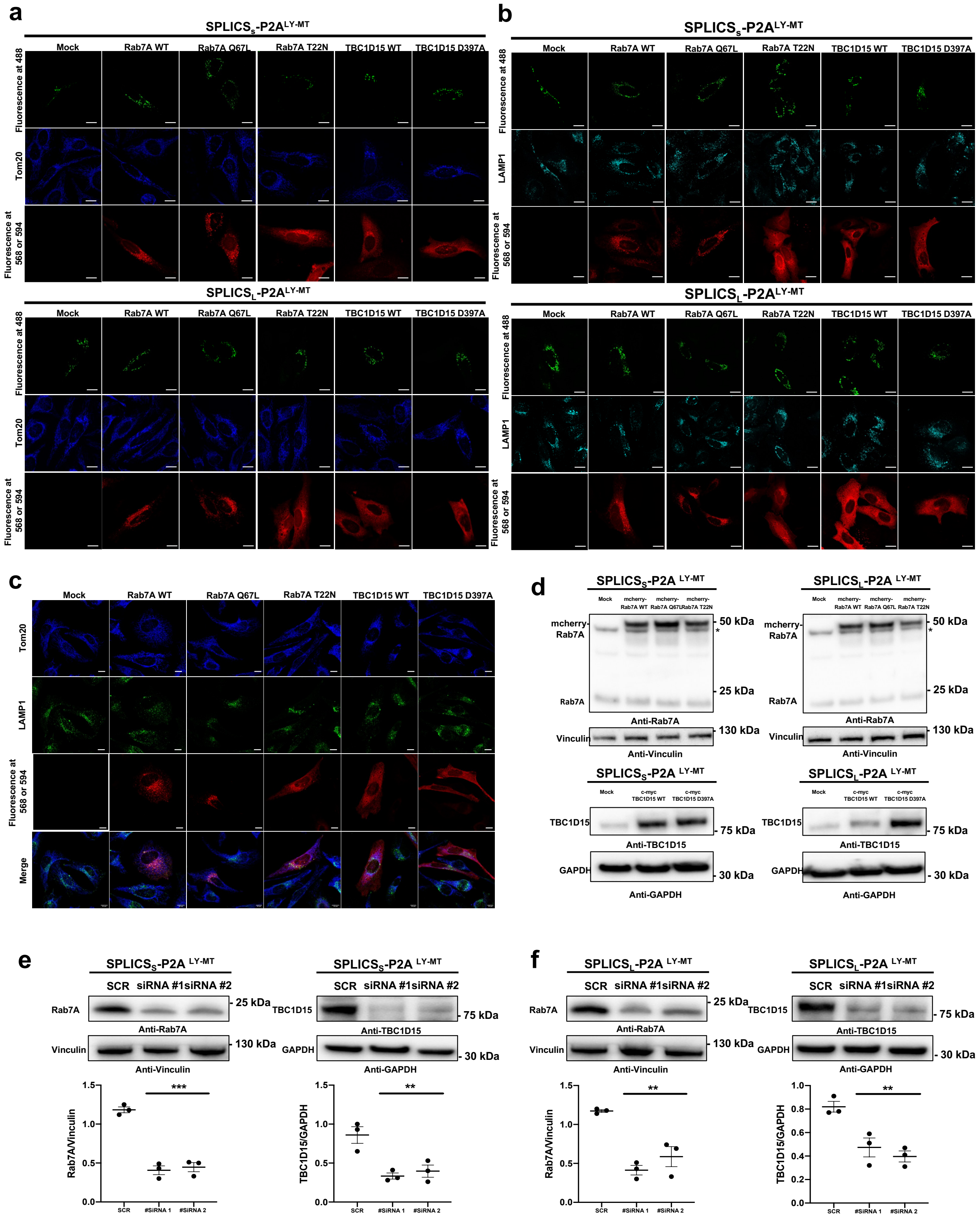

Supplementary Figure 1 Characterization of the Ly–Mt reporter. a-b Representative Z-projection images of HeLa cells transfected with SPLICS$_{S/L}$- P2A^{LY-MT} alone or plus Rab7A WT or Rab7A Q67L or Rab7A T22N or TBC1D15 WT or TBC1D15 D397A. Single channel of merged image reported in Fig. 2. SPLICS$_{S/L}$- P2A^{LY-MT} were represented by fluorescence "dots" upon excitation at 488 nm, mcherry -Rab7A WT or -Rab7A Q67L or -Rab7A T22N were represented upon excitation at 568 nm while c-myc -TBC1D15 WT or -TBC1D15 D397A by anti-cmyc upon excitation at 594 nm, mitochondria were detected by anti-Tom20 upon excitation at 405 nm. **b** Representative Z-projection images of HeLa cells transfected with SPLICS$_{S/L}$- P2A^{LY-MT} alone or plus Rab7A WT or Rab7A Q67L or Rab7A T22N or TBC1D15 WT or TBC1D15 D397A. Single channel of merged image reported in Fig. 2. SPLICS$_{S/L}$- P2A^{LY-MT} were represented by fluorescence "dots" upon excitation at 488 nm, mcherry -Rab7A WT or -Rab7A Q67L or -Rab7A T22N were represented upon excitation at 568 nm while c-myc -TBC1D15 WT or -TBC1D15 D397A by anti-cmyc upon excitation at 594 nm, lysosomes were detected by anti-LAMP1 upon excitation at 405 nm. **c** Representative Z-projection images of HeLa cells transfected with Rab7A WT or Rab7A Q67L or Rab7A T22N or TBC1D15 WT or TBC1D15 D397A. Mcherry -Rab7A WT or -Rab7A Q67L or -Rab7A T22N were represented upon excitation at 568 nm while c-myc -TBC1D15 WT or -TBC1D15 D397A by anti-cmyc upon excitation at 594 nm, mitochondria were detected by anti-Tom20 upon excitation at 405 nm and lysosomes were detected by anti-LAMP1 upon excitation at 488 nm. **d** Expression levels of overexpressed mcherry -Rab7A WT or c-myc -TBC1D15 WT were analyzed by Western blotting with anti-Rab7A or anti-TBC1D15 in HeLa cells transfected with SPLICS$_{S/L}$- P2A^{LY-MT} alone or plus Rab7A WT or Rab7A Q67L or Rab7A T22N or TBC1D15 WT or TBC1D15 D397A. Equal amount of total loaded lysate was verified by incubation with anti- vinculin or anti-GAPDH antibodies. * is aspecific band. **e-f** Expression levels of endogenous Rab7A WT or TBC1D15 WT were analyzed by Western blotting with anti-Rab7A or anti-TBC1D15 in HeLa cells transfected with SPLICS$_{S/L}$- P2A^{LY-MT} plus scramble or siRNA Rab7A #1 or siRNA Rab7A #2 or siRNA TBC1D15 #1 or siRNA TBC1D15 #2. Equal amount of total loaded lysate was verified by incubation with anti- vinculin or anti-GAPDH antibodies, mean ± SEM. Scale bar 10 μm. The data were obtained from three independent transfections. (**$p \leq 0.01$, ***$p \leq 0.001$ one-way ANOVA)

Supplementary Figure 2

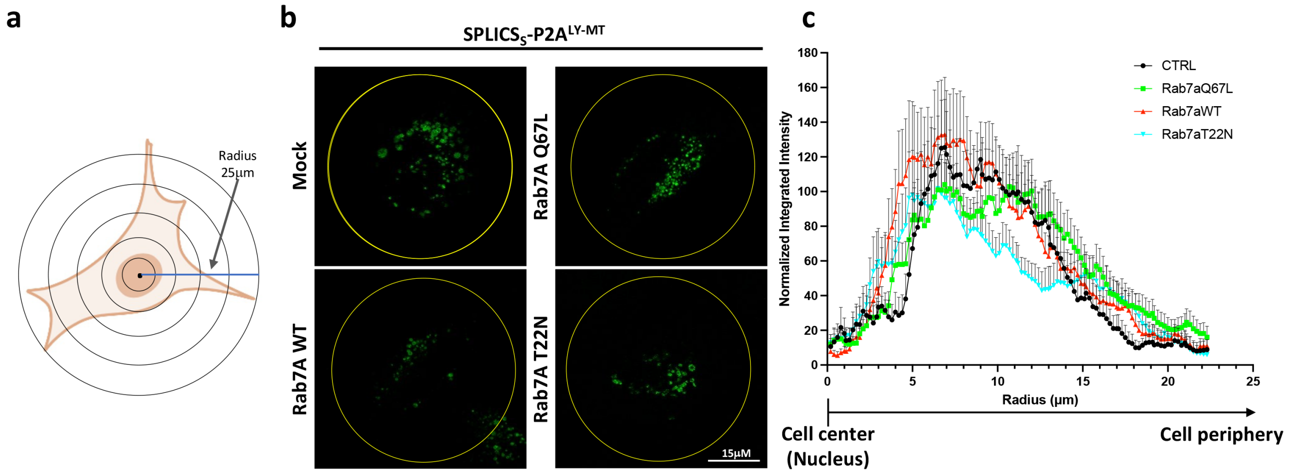

Supplementary Figure 2 Radial analysis of the SPLICS signal. a. Cartoon showing an example of the radial fluorescence analysis of cellular zones from the center of the nucleus to the cell periphery**. b**. Radial analysis (profile plot of normalized integrated intensities around concentric circles as a function of 25µm distance from the center of the cell nucleus) of HeLa cells expressing SPLICSs-P2A$^{LY\text{-}MT}$ either alone or in combination with the indicated Rab7 expression plasmids was performed as in [91] by using the ImageJ plgin Radial_Profile.class. The yellow circle has a radius of 25 µm. The normalized integrated intensity of the SPLICS signal over the radius in µm, mean ± SEM from the center of the nucleus is shown in **c**. The data were obtained from two independent transfections.

Supplementary Figure 3

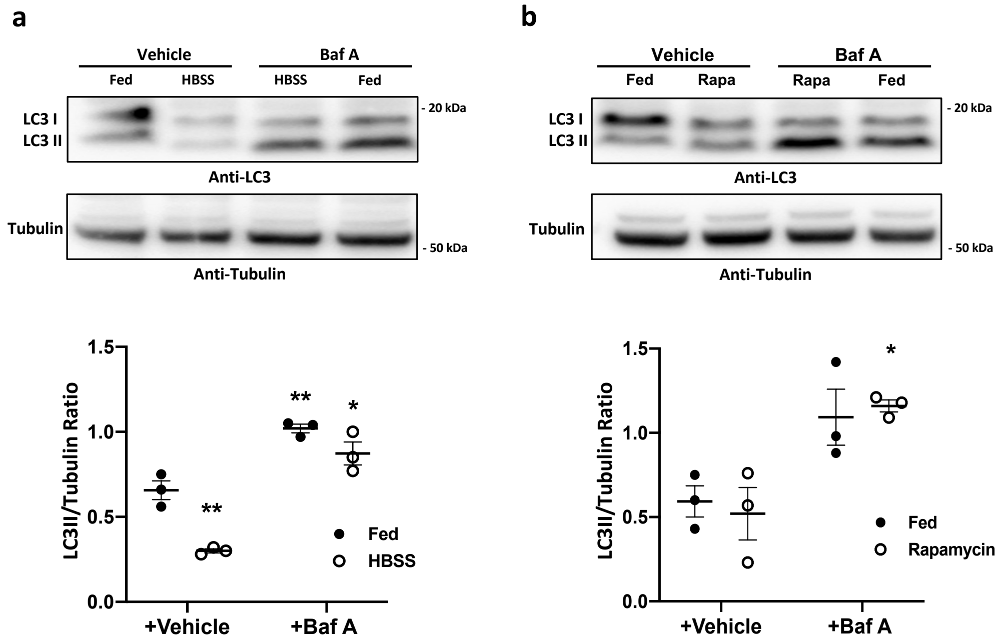

Supplementary Figure 3 Evaluation of starvation induction. a Expression levels of endogenous LC3I/LC3II were analyzed by Western blotting with anti-LC3 in HeLa cells untreated (Fed) or incubated in HBSS for 3h, in presence of Bafilomycin A (Baf A) or DMSO (Vehicle). Equal amount of total loaded lysate was verified by incubation with anti-β tubulin antibody. The graphs represent the densitometric quantification of LC3II signal over the tubulin signal (LC3II/tubulin Ratio), mean ± SEM. **b** Expression levels of endogenous LC3I/LC3II were analyzed by Western blotting with anti-LC3 in HeLa cells untreated (Fed) or incubated with1 μM rapamycin for 5h, in presence of Bafilomycin A (Baf A) or DMSO (Vehicle). Equal amount of total loaded lysate was verified by incubation with anti-β tubulin antibody. The graph represents the densitometric quantification of LC3II signal over the tubulin signal (LC3II/tubulin Ratio), mean ± SEM.

The data were obtained from three independent transfections. (*$p \leq 0.05$, **$p \leq 0.01$ unpaired two-tailed t-test)

Supplementary Figure 4

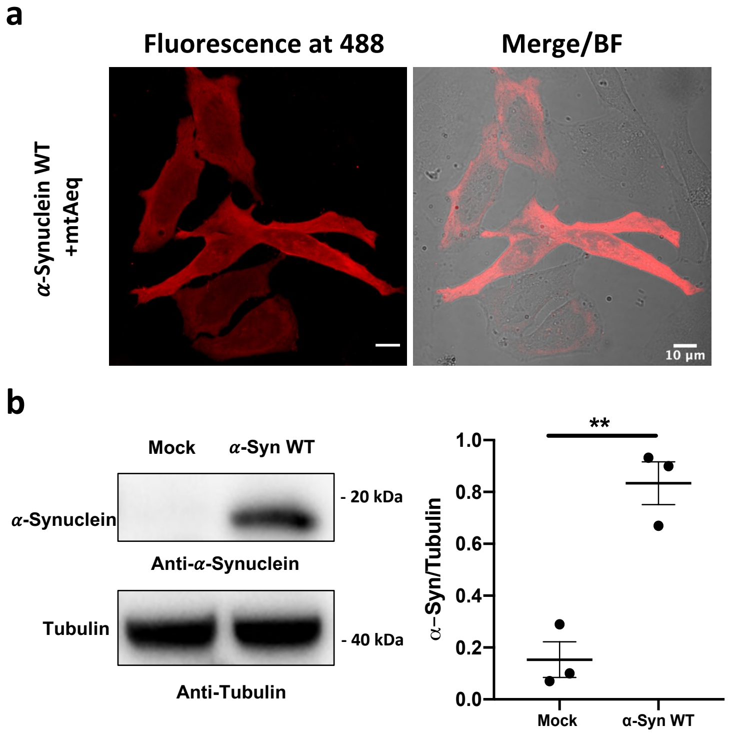

Supplementary Figure 4 Evaluation of α-synuclein overexpression. **a** Representative Z-projection images of HeLa cells co-transfected with mtAeqwt and α-Syn WT. α-synuclein was detected by anti-α-synuclein upon excitation at 633 nm (red) and merged with bright fields (BF). **b** Expression levels of overexpressed α-synuclein was analyzed by Western blotting with anti- α-synuclein in HeLa cells transfected with mtAeqwt alone (Mock) or plus α-Syn WT. Equal amount of total loaded lysate was verified by incubation with anti- β tubulin antibody. The graph represents the densitometric quantification of α-Syn/tubulin signal, mean ± SEM. Scale bar 10 μm. The data were obtained from three independent transfections. (**$p = 0.01$ unpaired two-tailed t-test)

Supplementary Figure 5

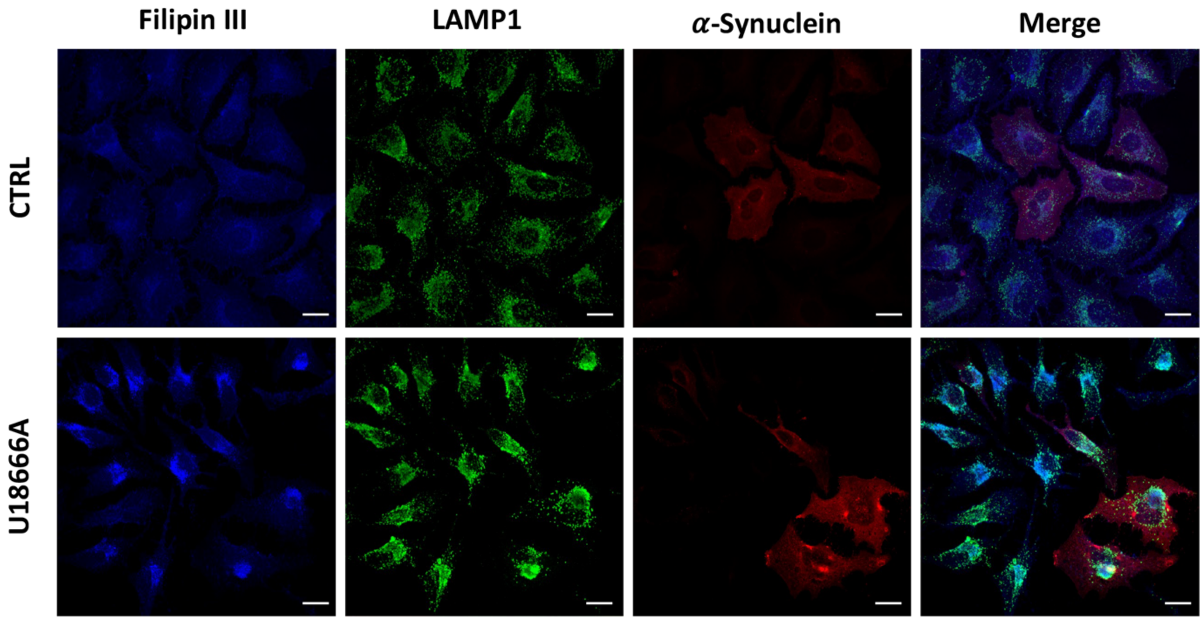

Supplementary Figure 5 α-synuclein overexpression does not impinge on U1866A-induced cholesterol accumulation. Representative Immunofluorescence of control HeLa cells and cells transfected with α-Syn WT. Filipin III staining was detected upon excitation at 380nm (blue), LAMP1 was detected by anti-LAMP1 upon excitation at 488nm (green), α-synuclein was detected by anti- α-synuclein upon excitation at 633 nm (red). Scale bar 10 µm. The data were obtained from three independent transfections.

Supplementary Figure 6

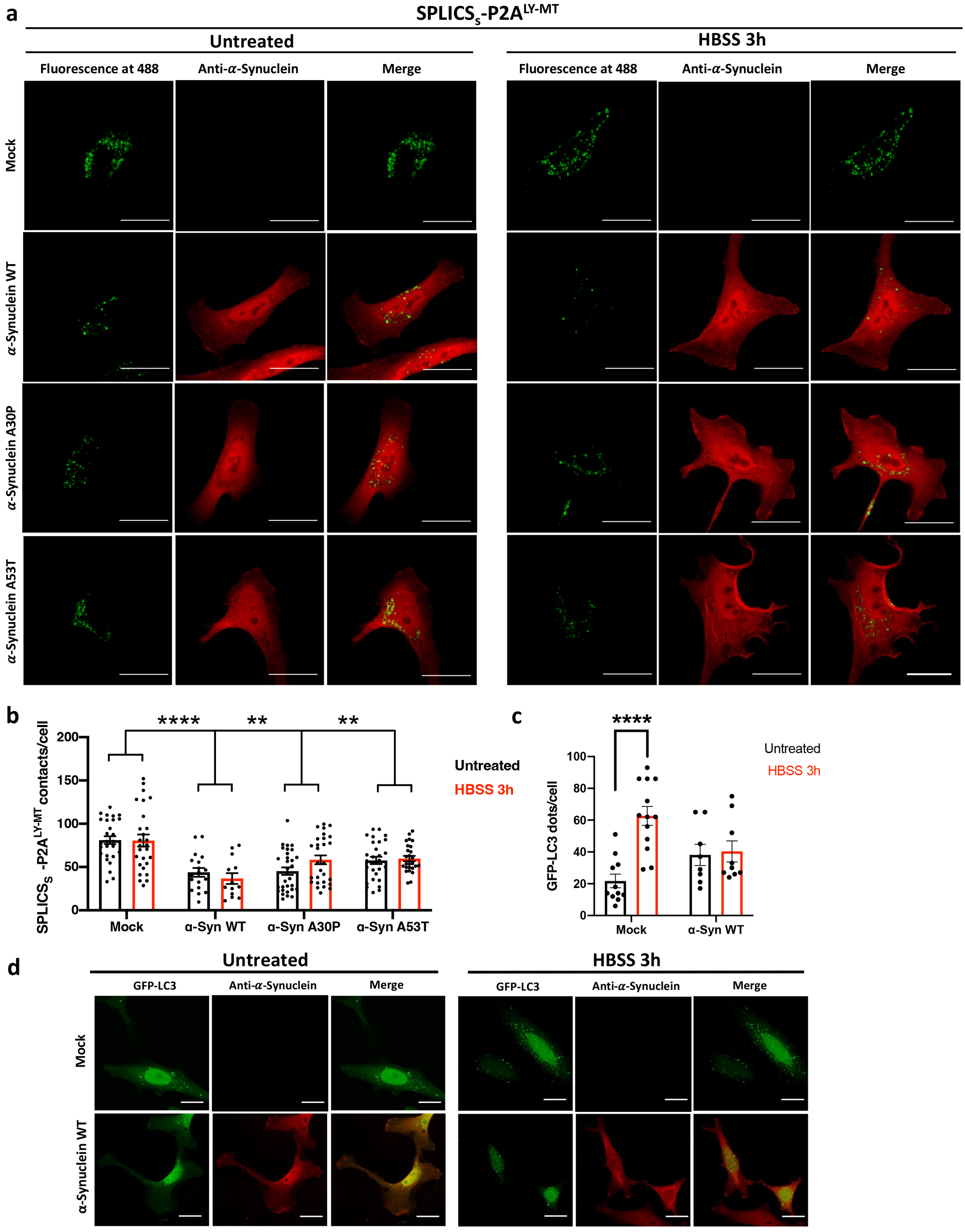

Supplementary Figure 6 α-Synuclein overexpression counteracts Ly-Mt short interactions under starvation. a Representative Z-projection images of HeLa cells transfected with SPLICS$_S$-

P2A^{LY-MT} (Mock) or co-transfected with SPLICS$_S$- P2A^{LY-}MT and α-Syn WT or α-Syn A30P or α-Syn A53T in untreated condition or under HBSS treatment. SPLICS$_S$- P2A^{LY-MT} was represented by fluorescent "dots" upon excitation at 488 nm (green) and α-synuclein was detected by anti- α-synuclein upon excitation at 633 nm (red). Merge panels are shown. **b** Quantification of short LY-MT contacts (Mean ± SEM: Untreated: SPLICSS-P2A^{LY-MT} + Mock 81.05 ± 4.54 n=28; SPLICSS-P2A^{LY-MT} + WT 43.83 ± 5.05 n=18 ****p ≤ 0.0001; SPLICSS-P2A^{LY-MT} + A30P 45.26 ± 4.27 n=31**p ≤ 0.01; SPLICSS- P2A^{LY-MT} + A53T 57.59 ± 4.25 n=28 **p ≤ 0.01. HBSS treated: SPLICSS- P2A^{LY-MT} + Mock 80.57 ± 7.06 n=27; SPLICSS-P2A^{LY-MT} + WT 38.80 ± 7.17 n=15 ****p ≤ 0.0001; SPLICSS-P2A^{LY-MT} + A30P 58.39 ± 5.09 n=28 **p ≤ 0.01; SPLICSS-P2A^{LY-MT} + A53T 59.71 ± 3.19 n=26 **p ≤ 0.01). **c** Quantification of GFP-LC3 dots per cell in untreated or HBSS treated cells co-transfected with pcDNA3 (Mock) or α-Syn WT and GFP-LC3 (Mean ± SEM: Mock Untreated 21.72 ± 4.28 n=11 vs HBSS treated 62.69 ± 5.95 n=13 ****p ≤ 0.0001; α-Syn WT Untreated 38.12 ± 6.63 n=8 vs HBSS treated 40.33 ± 6.61 n=9). **d** Representative Z-projection images of HeLa cells untreated or HBSS treated overexpressing pcDNA3 (Mock) or α-SYN WT in presence of GFP-LC3. The SPLICS$_S$- P2A^{LY-MT} contacts were quantified from the 3D rendering of a complete Z-stack, mean ± SEM. Scale bar 10 μm. The data were obtained from three independent transfections. (**p ≤ 0.01, ****p ≤ 0.0001 two-way ANOVA)

Full Scan for Supplementary Information

Rab7a and TBC1D15 Overexpression
Supplementary Figure 1**d**

Rab7a SPLICS$_s$ and SPLICS$_L$

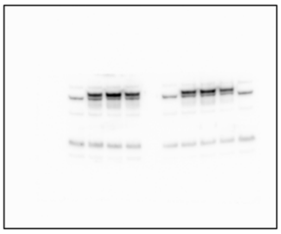

TBC1D15 SPLICS$_s$ and SPLICS$_L$

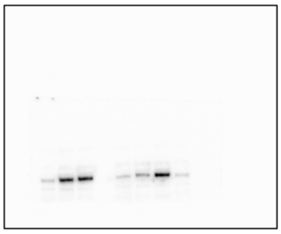

Vinculin SPLICS$_s$ and SPLICS$_L$

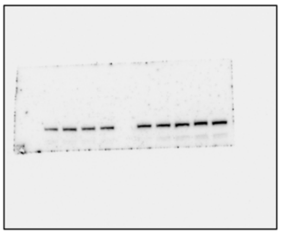

GAPDH SPLICS$_s$ and SPLICS$_L$

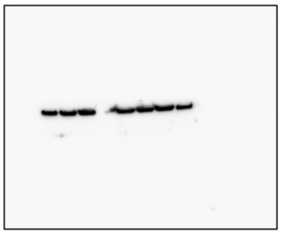

Rab7a and TBC1D15 SiRNA
Supplementary Figure 1**e** and **f**

Rab7a SPLICS$_s$ and SPLICS$_L$

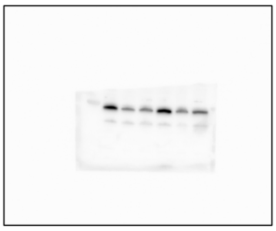

TBC1D15 SPLICS$_s$ and SPLICS$_L$

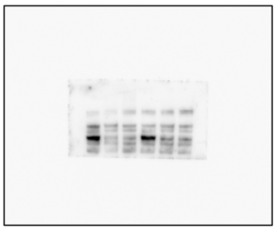

Vinculin SPLICS$_s$ and SPLICS$_L$

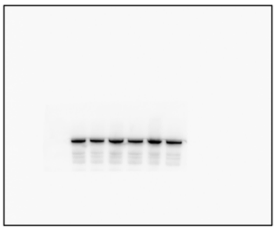

GAPDH SPLICS$_s$ and SPLICS$_L$

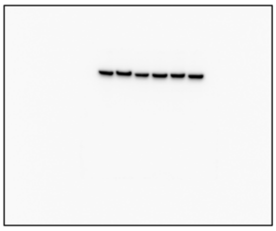

LC3
Supplementary Figure 3**a** and **b**

LC3 (HBSS)

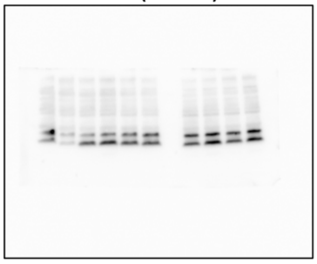

LC3 (Rapa)

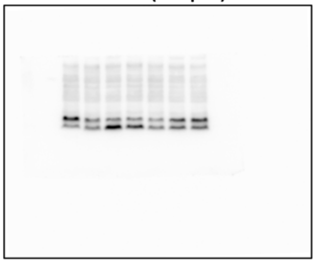

Tubulin (HBSS)

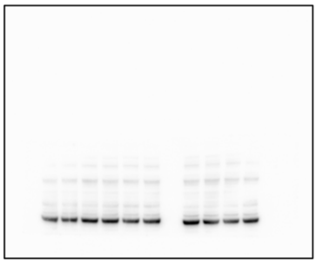

Tubulin (Rapa)

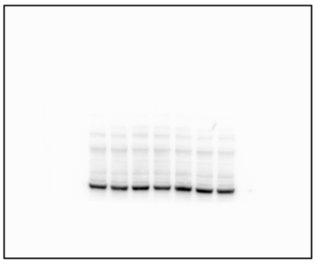

Synuclein
Supplementary Figure 4**b**

Alpha-Synuclein

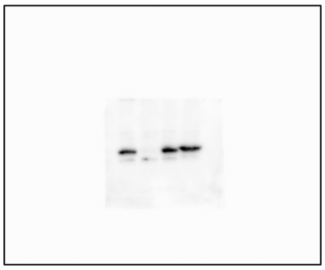

Tubulin

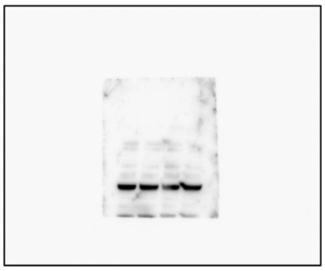

Supplement: Supplementary file 1 — Supplementary Information [file 41467_2024_46007_MOESM1_ESM.pdf]
